# Comparison of the Effects of Enzymolysis Seaweed Powder and *Saccharomyces boulardii* on Intestinal Health and Microbiota Composition in Kittens

**DOI:** 10.3390/metabo13050637

**Published:** 2023-05-08

**Authors:** Mingrui Zhang, Ruixia Mo, Mingtan Li, Yuankai Qu, Haotian Wang, Tianyi Liu, Pan Liu, Yi Wu

**Affiliations:** 1 State Key Laboratory of Animal Nutrition, College of Animal Science and Technology, China Agricultural University, Beijing 100193, China; 2 Shidai Marine Biotechnology Co., Ltd., Weihai 264319, China

**Keywords:** enzymolysis seaweed, *Saccharomyces boulardii*, kitten, intestinal health, gut microbiota

## Abstract

Kittens are prone to intestinal health problems as their intestines are not completely developed. Seaweed is rich in plant polysaccharides and bioactive substances that are highly beneficial to gut health. However, the effects of seaweed on cat gut health have not been assessed. This study compared the effects of dietary supplementation with enzymolysis seaweed powder and *Saccharomyces boulardii* on the intestinal health of kittens. In total, 30 Ragdoll kittens (age: 6 months; weight: 1.50 ± 0.29 kg) were assigned to three treatment groups for a 4-week feeding trial. The dietary treatment given was as follows: (1) basal diet (CON); (2) CON + enzymolysis seaweed powder (20 g/kg of feed) mixed evenly with the diet (SE); and (3) CON + *Saccharomyces boulardii* (2 × 10^10^ CFU/kg of feed) mixed evenly with the diet (SB). Compared with the CON and SB groups, dietary supplementation with the enzymolysis seaweed powder improved the immune and antioxidant capacity and also reduced the intestinal permeability and inflammation levels of kittens. The relative abundance of Bacteroidetes, Lachnospiraceae, Prevotellaceae, and *Faecalibacterium* in the SE group was higher than those in the CON and SB groups (*p* ≤ 0.05), while the relative abundance of Desulfobacterota, Sutterellaceae, and *Erysipelatoclostridium* in the SB group was lower than that in the SE group (*p* ≤ 0.05). Moreover, enzymolysis seaweed powder did not alter the level of intestinal SCFAs in kittens. Conclusively, supplementing kitten diet with enzymolysis seaweed powder can promote intestinal health by enhancing the gut barrier function and optimizing the microbiota composition. Our findings provide new perspectives on the application of enzymolysis seaweed powder.

## 1. Introduction

Gut health determines the whole health of the animal because the gut is not only a vital organ for digestion and nutrient absorption but also an immune organ [1]. Kittens have underdeveloped gut; therefore, they are more prone to intestinal health problems and exhibit symptoms of vomiting, diarrhea, reflux, and weight loss [2]. Notably, intestinal health in early life is critical to health and disease development in later life [3]. The colonization of gut microbes in infancy may define the lifelong composition of the microbiome [4]. Moreover, gut microbes influence nutritional intake, regulate intestinal homeostasis and physiological function, and drive immune responses in pets, thereby affecting their health [5]. Nutritional interventions in the diet can rapidly and repeatedly modify the composition and function of the gut microbiota [6]. Numerous studies have indicated that altering the dietary composition of cats can significantly influence the gut microbiome within 1 to 2 weeks [7,8]. There were significant differences in the composition of the intestinal microbiota between young and adult cats, and a marked increase in the structural and functional diversity of microbes was reported in adult cats [9]. Therefore, optimizing the intestinal microbiota structure through dietary interventions may be an essential means of improving the gut health of kittens. However, very few studies have used kittens as test subjects, with little data available for reference. Current studies have generally been conducted on adult cats.

Prebiotic supplementation is currently among the significant methods for improving the intestinal health of pets. Seaweed is rich in various bioactive compounds with anticancer, antioxidant, anti-inflammatory, and antibacterial properties [10,11,12]. More importantly, most of these active substances can resist gastric acid and host digestion, making seaweed a promising substrate for prebiotics [13]. Seaweed and its extracts can enhance nutrient digestibility and immune system function, thereby improving the growth and production performance of economic animals [14,15,16]. Furthermore, studies have shown that polysaccharides in seaweed can scavenge free radicals and enhance the endogenous antioxidant system of animals, thereby treating various diseases [17]. Meanwhile, seaweed polysaccharides are responsible for anticancer effects through inducing apoptosis of cancer cells, inhibiting lysosome-dependent autophagy, and suppressing cancer cell invasion and metastasis [18]. Conversely, research on the impacts of seaweed on pets is limited. A study showed that dogs fed a seaweed-supplemented diet exhibited no change in nutrient digestibility, fecal microbiota, and the metabolome [19]. Moreover, studies on the effects of seaweed on cat intestinal health have not yet been conducted.

Probiotics alter the host gut microbiota through different mechanisms, such as by changing the abundance of pathogenic bacteria, promoting microbiota interactions to stimulate the growth of resident bacteria, or indirectly influencing the microbiota structure through metabolite secretion [20]. Notably, probiotics are effective only if they tolerate stomach acid and bile salts, and adhere to and colonize the intestinal lumen. Otherwise, they will be excreted as passers-by bacteria. Factors such as resident microbiota, host characteristics, and diet determine the colonization success of probiotics [21]. In addition, some studies have questioned whether probiotics are safe for use in pets [22]. Therefore, additional studies are required to track the benefits of probiotics as well as the frequency and severity of their adverse outcomes in pets over time. Accumulating evidence suggests that *Saccharomyces boulardii* (*S. boulardii*) can inhibit the colonization of pathogenic microorganisms, improve intestinal barrier function, and regulate immunity [23]. This microbe is widely used as a commercial probiotic in pets. *S. boulardii* supplementation can improve the intestinal status, reduce stress, and act as an effective adjunct to the treatment of chronic enteropathies in dogs [24,25]. However, studies on the application of *S. boulardii* in cats are few, and its effects on the intestinal health of cats and the underlying action mechanisms remain unclear.

Ragdoll kittens were selected in this study because they have delicate intestines and a higher incidence of intestinal diseases. The study compared the effects of the enzymolysis seaweed powder and *S. boulardii* on intestinal health in kittens, particularly focusing on the gut barrier function and microbiota composition.

## 2. Materials and Methods

Experimental protocols were approved by the Institutional Animal Care and Use Committee of China Agricultural University (AW31402202-1-6).

### 2.1. Animals and Experimental Treatments

Before the start of the research, a medical examination was performed on a colony of Ragdoll kittens, which included blood and serum analyses, urinalysis, appetite, body condition, fecal score, and parasite. Thirty (half male and half female) Ragdoll kittens (6-month old; weighed 1.50 ± 0.29 kg) with mild diarrhea were selected. The body condition score of all kittens was between 4 and 5 points (on a 9-point scale) [26], and their fecal score was between 3.5 and 4.5 points (on a 5-point scale) [27]. None of the kittens had immune-mediated diseases, allergies, or other conditions causing chronic gastrointestinal dysfunction. For 3 months before the trial, the kittens were not given any function-related drugs, and they were kept in the same feeding environment with the same basal diet. Thirty cats in good health were fully involved in the trial.

Thirty kittens were randomly assigned to three treatment groups with 10 replicates per group, including 5 males and 5 females. The dietary treatment given was as follows: (1) basal diet (CON); (2) CON + enzymolysis seaweed powder (20 g/kg of feed, SE); and (3) CON + *S. boulardii* (2 × 10^10^ CFU/k g of feed, SB). The basal diet was a dry extruded diet, which met the nutritional requirements recommended by the NRC (2006), and its raw material composition and nutritional level were close to those of commercially available cat food (Appendix A Table A1). Enzymolysis seaweed and *S. boulardii* were both powders, and evenly sprinkled and attached to the surface of the dry extruded diet. The enzymolysis seaweed powder (Shidai Marine Biotechnology Co., Ltd., Weihai, China) was obtained by hydrolyzing a *Laminaria* spp. with complex enzymes, containing 7.8% laminarin and 69% ash. *S. boulardii* (Levucell SB) was obtained from the Lallemand Animal Nutrition Group (Montreal, Canada). The experiment lasted for 28 days. The feed consumption of each kitten was recorded daily. Body weights (BWs) and fecal scores in each treatment were recorded on d 0, 14, and 28 to evaluate growth status and fecal quality, respectively.

### 2.2. Sample Collection

On d 28, 1 mL of blood was drawn from the forelimb head vein of each kitten into a vacutainer containing heparin sodium, centrifuged at 3000 r/min for 15 min at 4 °C to separate plasma, and stored at −20 °C for short-term preservation and further analysis. On d 28, a sterilized tray was placed under each cage to hold kitten feces; 5 g of fresh feces from each kitten was collected separately in sterile tubes with sterilized forceps, avoiding contact with other surfaces or materials. The collected fecal samples were preserved at −80 °C for microbiota and SCFA analyses.

### 2.3. Plasma Parameters Measurement

The plasma concentrations of immunoglobulins (IgA and IgG), inflammatory factors interleukin-1β (IL-1β), IL-6, IL-10, and tumor necrosis factor-α (TNF-α) were analyzed using commercially available ELISA kits following the manufacturer’s instructions. Plasma superoxide dismutase (SOD) and malondialdehyde (MDA) levels that determine the antioxidant capacity were also assessed using ELISA kits according to the manufacturer’s instructions. The concentrations of d-lactate (D-LA), lipopolysaccharide (LPS), diamine oxidase (DAO), and intestinal fatty acid-binding protein (iFABP) were determined using ELISA kits following the manufacturer’s protocols. All commercial kits were sourced from Shanghai Enzyme-linked Biotechnology Co., Ltd. (Shanghai, China).

### 2.4. Fecal Microbiota Analysis

The QIAamp Fast DNA Stool Mini Kit (Qiagen, Germany) was used for the extraction of total bacterial DNA in kitten feces. The V3–V4 region of the 16S rRNA was amplified using universal primers 338F (5′-ACTCCTACGGGAGGCAGCAG-3′) and 806R (5′-GGACTACHVGGGTWTCTAAT-3′), subsequently pooled into equimolar amounts, and sequenced on the Illumina MiSeq platform to generate paired-end reads of 300 base pairs (bp). The fastp software (version 0.19.6) was used for quality control of the raw sequences of 16s rRNA, and FLASH software (version 1.2.11) was used for splicing. Then the optimized sequences were clustered into operational taxonomic units (OTUs) using UPARSE software (Version 11) with 97% sequence similarity level. The taxonomy of each OTU representative sequence was analyzed by the RDP Classifier (Version 2.2) against the Silva 138 database using a confidence threshold of 0.7. Alpha diversity indices (the Shannon index and Simpson index) were calculated with Mothur (Version 1.30.2). Beta diversity was analyzed using Bray Curtis distance and visualized by principal component analysis (PCA). The community species composition of each sample was determined at each taxonomic level. The Kruskal–Wallis H test was used to detect significant differences in abundance among the groups. Raw data were uploaded to the NCBI SRA Database with accession number SRP422749. 

### 2.5. Determination of Fecal SCFAs

The SCFA concentrations in the feces were determined through GC-MS (Shanghai Major Biomedical Technology Co., Ltd., Shanghai, China). Briefly, methanol and the standard of each SCFA were used to prepare SCFA standard working solutions at different concentrations (10, 50, 100, 200, 300, 400, and 500 μg/mL) for GC-MS detection and analysis to obtain the standard curves of each SCFA. After that, 100 mg of feces was weighed, added to 450 μL methanol and 50 μL 2-ethylbutyric acid (1000 μg/mL) for 30 min of sonication in an ice bath, and then centrifuged at 13,000× *g* for 15 min at 4 °C to separate the supernatant. Next, 50 mg of anhydrous sodium sulfate was added to the supernatant, and the supernatant was centrifuged at 13,000× *g* for 15 min at 4 °C after the vortex. The supernatant solution was collected and analyzed using the 8890B-5977B GC/MSD (Agilent Technologies Inc., Santa Clara, CA, USA). 

### 2.6. Statistical Analysis

Data analysis was performed using IBM SPSS Statistics 26 (Chicago, IL, USA) and GraphPad Prism (version 8.3.0, San Diego, CA, USA). Microbial sequencing data were graphed using the R tool. The bar graphs of the bacterial community were prepared using the R ggplot package, and heatmaps were constructed using the R vegan package. The abundance of microbiota and fecal scores were compared using the Kruskal–Wallis H test. Other data were compared through one-way ANOVA using the Tukey post-hoc test. A score of *p* ≤ 0.05 was considered statistically significant. Data are expressed as the mean ± SEM.

## 3. Results

### 3.1. Growth Performance

Table 1 presents the effects of different dietary treatments on the growth performance of kittens. No significant differences in the average daily feed intake and the BW of kittens were observed on d 0, 14, and 28 among the three treatments (*p* > 0.05). However, compared with kittens that consumed CON and SB diets, the ADG of kittens that consumed the SE diet significantly increased during d 15 to 28 (*p* ≤ 0.05). Additionally, on d 0 to 28, ADG in the SE group was greater than that in the CON group (*p* ≤ 0.05). However, ADG did not differ significantly between the SE and SB groups or the SB and CON groups (*p* > 0.05).

### 3.2. Fecal Score

Table 2 presents the effects of different dietary treatments on fecal scores. Because of our screening of experimental kittens, no significant difference was observed in the fecal scores among the three groups on d 0. On d 14, the fecal score of kittens in the SE group was lower than that in the CON group (*p* ≤ 0.05). However, the score did not differ significantly between the SE and SB groups or the SB and CON groups (*p* > 0.05), but, compared with kittens that consumed the CON diet, the fecal score of kittens that consumed with SE and SB diets was significantly decreased on d 28 (*p* ≤ 0.05).

### 3.3. Immunoglobulin and Antioxidant Parameters

Table 3 presents the immunoglobulin and antioxidant parameters of kittens subjected to different treatments. The IgA and IgG levels were significantly higher in the SE group than those in the CON and SB groups (*p* ≤ 0.05) but significantly lower in the CON group than those in the SB group (*p* ≤ 0.05). Compared with kittens that consumed CON and SB diets, the SOD concentration significantly increased and the MDA concentration significantly decreased in kittens that consumed the SE diet (*p* ≤ 0.05). The SB group had higher (*p* ≤ 0.05) SOD concentration and lower (*p* ≤ 0.05) MDA concentration compared with the CON group. 

### 3.4. Inflammatory Factors

As shown in Table 4, the SE group exhibited increased serum IL-10 levels compared with the CON and SB groups (*p* ≤ 0.05). Meanwhile, compared with the CON and SB groups, IL-1β, IL-6 and TNF-α levels decreased in the SE group (*p* ≤ 0.05). Moreover, serum IL-10 levels increased but IL-1β, IL-6 and TNF-α levels decreased in the SB group compared with those in the CON group (*p* ≤ 0.05).

### 3.5. Gut Barrier Function Parameters

The gut barrier function parameters of kittens in the different dietary treatment groups are listed in Table 5. The D-LA, LPS, DAO, and iFABP levels were decreased in the SE group compared with those in the CON and SB groups (*p* ≤ 0.05), and were decreased in the SB group compared with those in the CON group (*p* ≤ 0.05).

### 3.6. Fecal Microbiota Composition

**Figure 1:**
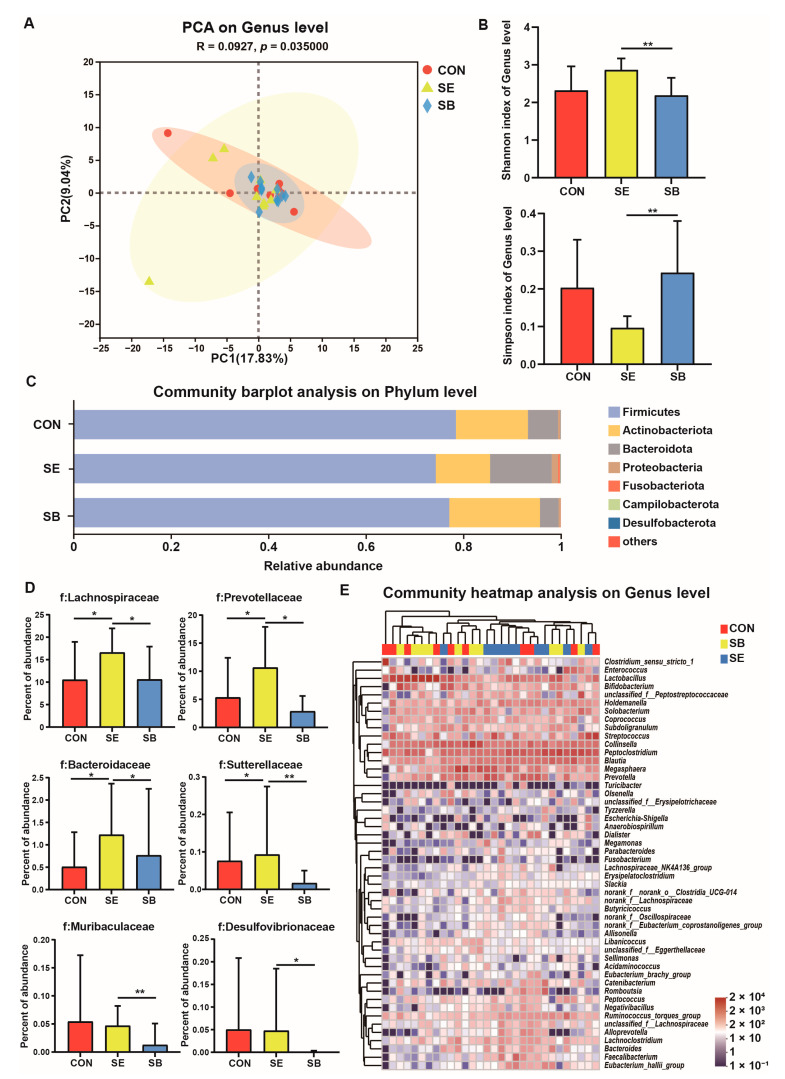
Changes in the microbial structure in feces of kittens subjected to different treatments. (**A**) Principal component analysis at the genus level. (**B**) The α-diversity analysis using the Shannon and Simpson indices. (**C**) The relative abundance of fecal bacteria at the phylum level. (**D**) The relative abundance of Lachnospiraceae, Prevotellaceae, Bacteroidaceae, Sutterellaceae, Muribaculaceae, and Desulfovibrionaceae. (**E**) The relative abundance of fecal bacteria at the genus level. CON, basal diet; SE, basal diet + enzymolysis seaweed powder (20 g/kg of feed); SB, basal diet + *S. boulardii* (2 × 10^10^ CFU/kg of feed). ** *p* < 0.01, * *p* < 0.05.

Figure 1 presents changes in the microbiota composition in kitten feces after different treatments. PCA demonstrated distinct clustering of fecal microbial communities for each group (*p* ≤ 0.05; Table A1). Compared with the SB group, the SE group had a higher Shannon index and a lower Simpson index (*p* ≤ 0.05; Figure 1B). This indicated that SE consumption increased microbial richness and diversity. Furthermore, we identified fecal bacteria at the phylum, family, and genus levels, exhibiting differences in the abundance across groups. At the phylum level (Figure 1C), the abundance of Bacteroidetes in the fecal microbiota of the SE group was higher than that of the CON and SB groups (*p* ≤ 0.05). The abundance of Desulfobacterota was significantly lower in the SB group than that in the CON and SE groups (*p* ≤ 0.05). At the family level (Figure 1D), the abundance of Lachnospiraceae, Prevotellaceae, and Bacteroidaceae was higher in the SE group than that in the CON and SB groups (*p* ≤ 0.05). The abundance of the Muribaculaceae was significantly increased in the SE group compared with the SB group (*p* ≤ 0.05). The fecal microbiota of the SB group had lower abundance of Sutterellaceae and Desulfovibrionaceae compared with that of the SE group (*p* ≤ 0.05). At the genus level (Figure 1E), the abundance of *Faecalibacterium*, *Bacteroides*, and *Eubacterium_hallii_group* increased in the SE group compared with those in the CON and SB groups (*p* ≤ 0.05). The abundance of *Erysipelatoclostridium* and *Flavonifractor* in the fecal microbiota of the SB group was lower than that of the SE group (*p* ≤ 0.05); however, it did not differ significantly between the CON and SB groups (*p* > 0.05). The relative abundance of *Sutterella* was lower in the SB group than that in the CON and SE groups (*p* ≤ 0.05).

### 3.7. Fecal SCFAs

As shown in Table 6, there were no significant differences in fecal concentrations of acetate, propionate, butyrate, isobutyrate, valerate, and isovalerate among the three treatment groups (*p* > 0.05). 

## 4. Discussion

The intestinal development of young animals is not sound, and they lack a stable intestinal microbiota, which makes them prone to intestinal microecology disorders. It is well known that dietary supplementation with probiotics and prebiotics is beneficial to animal intestines and, therefore, related products have been used in pet food. In vivo studies have focused on revealing the action mechanisms of specific dietary components. However, because of the large individual differences, the number of in vivo trials conducted on pets is very low, and adult animals are commonly used for experiments. In this study, it will be of special significance to take the lead in using kittens with larger scale and better sample uniformity. Numerous studies have shown that enzymolysis seaweed powder has a crucial application value in the breeding of economic animals and positively influences the production performance and body health of livestock and poultry [14,28,29]. Unfortunately, no application research has been conducted on enzymolysis seaweed powder in cats. Therefore, to provide data supporting the application of enzymolysis seaweed powder in pet food, this study explored the effects of this seaweed powder on immunity, antioxidant properties, intestinal permeability, and microbiota composition of kittens compared with the common commercial *S. boulardii*.

Immunoglobulin is a globulin with antibody activity and is widely found in mammalian serum, interstitial fluid, and exocrine fluid. The plasma immunoglobulin level can indirectly reflect the animal’s ability to resist exogenous stimuli and pathogens. It is a critical parameter to evaluate the immunity of animals. As the major component of serum immunoglobulin, IgG accounts for 75–80% of total serum immunoglobulin. It is mainly synthesized and secreted by plasma cells in the spleen and lymph nodes [30]. IgG plays a pivotal role in combating infections in young animals [31]. IgA is crucial for local anti-infection action of the body’s mucosa. The blocking of IgA synthesis increases susceptibility to local infections with microorganisms [32]. Studies have shown that dietary supplementation with seaweed by-products increased IgA concentration in the serum of chicks [33]. Serum IgG concentration in rats supplemented with heat-treated dried brown seaweed increased significantly after 16 weeks [34]. These results are in line with our findings, which showed that direct dietary supplementation with enzymolysis seaweed powder increased IgA and IgG levels. In addition, supplementation with seaweed extract increased IgA and IgG levels in maternal blood and colostrum, further enhancing the circulating IgG concentration and the percentage of leukocytes and lymphocytes phagocytosing Escherichia coli in lactating juvenile animals. This suggested that enzymolysis seaweed powder could improve the immune function by increasing immunoglobulin levels in young animals [35,36].

A stable reactive oxygen species (ROS) is essential for maintaining a normal physiological function. Under normal circumstances, the free radical content is at an equilibrium level. These free radicals are involved in cell signal transduction, synthesis and metabolism of substances, and energy production in the body. However, oxidative stress can trigger disease development when an imbalance between ROS production and antioxidant networks occurs [37]. Owing to their high sensitivity and activity, cellular stress levels may be higher in young animals [38]. SOD scavenges superoxide free radicals, and MDA is a lipid peroxidation product generated in the metabolism of oxygen free radicals in the organism. In this study, compared with the basal diet and the diet supplemented with *S. boulardii*, the addition of enzymolysis seaweed powder to the diet significantly increased the SOD level and reduced the MDA level in kittens. Consistent with our findings, a study showed that seaweed polysaccharides may increase SOD, CAT, and GSH levels and decrease the MDA level by regulating the NRF2 signaling pathway [39]. In addition, seaweed polysaccharides exert a beneficial effect on acetaminophen-induced acute liver injury in rats by elevating GSH, GSH-Px, and SOD expression and decreasing the MDA concentration [40]. Notably, supplementation of seaweed-derived polysaccharides increased the SOD level of weaned piglets at 24 days of age, along with changes in intestinal microbiota and increase in acetic acid and butyric acid concentrations [41].

Studies have confirmed the role of gut microbes in contributing to growth and development, maintaining health, and regulating the occurrence and treatment of various diseases in pets [42,43]. Moreover, the intestinal microbes and their functional products affect host health [44,45]. Cats are obligate carnivores and need a protein-rich diet. Therefore, the gut microbiota composition of cats is different from those of humans and many other mammals. However, the amount of microbiome research conducted in cats is relatively small compared with that in humans and other mammals. *Firmicutes*, *Bacteroidetes*, *Actinobacteria*, *Proteobacteria*, and *Fusobacteria* were the predominant phyla in previous feline microbiota reports that used different sequencing technologies [7,46,47], and these results are consistent with those of our study. Aging in the gut is related to changes in microbial composition, developing from colonization in early life to relative stability in adulthood and then decreasing diversity in old age [7,9,48]. Bacteroides were less represented in the kitten intestines but significantly increased in the adult cats, probably balanced by a reduced abundance of *Firmicutes* and *Actinobacteria* [7,49]. In our study, enzymolysis seaweed powder increased the Shannon index, decreased the Simpson index, and increased the abundance of *Bacteroidetes*, indicating that enzymolysis seaweed powder is beneficial for the stability and maturity of kitten intestinal microbiota. The abundance of Prevotellaceae and Bacteroidaceae (members of the phylum Bacteroidetes) as well as Lachnospiraceae and *Faecalibacterium* (members of the phylum Firmicutes) was significantly reduced in dogs with chronic enteropathies [50,51,52,53]. These gut bacteria are known to produce SCFAs. In our study, enzymolysis seaweed powder added to the basal diet increased the abundance of these bacteria. In addition, Muribaculaceae, much like Bacteroides, ferment plant polysaccharides to produce propionate [54]. Consistent with the study findings, dietary addition with brown seaweed elevated the relative abundance of Muribaculaceae in the gut of high-fat- and high-sugar-diet-induced obese rats [55]. Although *Eubacterium hallii* cannot degrade complex polysaccharides and oligosaccharides, it can produce butyrate by using fermentation metabolites such as acetate and lactic acid [56]. Supplementing enzymolysis seaweed powder significantly increased the abundance of *Eubacterium_hallii_group*, suggesting that SCFA generation changes to some extent. Thus, according to these results, enzymolysis seaweed powder may promote intestinal health by increasing the abundance of beneficial bacteria. Moreover, the abundance of *Sutterella* significantly increased in dogs with acute diarrhea [51]. Meanwhile, the abundance of *Desulfovibrio* increased markedly in the intestines of cats with inflammatory bowel disease [57]. *Erysipelatoclostridium* and its related metabolite, ptilosteroid A, have been considered markers of radiation-induced intestinal damage [58]. In this study, dietary addition with *S. boulardii* markedly reduced the relative abundance of *Desulfovibrionaceae*, *Erysipelatoclostridium*, and *Sutterella*, suggesting that *S. boulardii* improves the gut microbiota structure in kittens by lowering harmful bacteria.

The SCFAs are the main metabolites of intestinal microorganisms and serve as a source of energy for gut epithelial cells. They have various biological functions, such as enhancing the gut mucosal barrier function and participating in intestinal immune regulation [59]. Acetic acid, propionic acid, and butyric acid are the most abundant SCFAs in the animal’s digestive tract. Similar to the case in other species, probiotics and prebiotics can affect intestinal SCFA production in felines by regulating the intestinal microbiota structure, especially by increasing the amount and activity of SCFA-producing bacteria [1]. The acetate and propionate contents of pre-digested red seaweed increased significantly after fermentation in a colonic model in vitro [60]. Furthermore, dietary supplementation with 400 mg/kg seaweed-derived polysaccharides significantly increased the cecal concentrations of acetate and butyrate in weaned piglets [41]. However, dietary addition with seaweed had no effect on the SCFA composition and concentration in dog feces [19], which is consistent with our findings in kittens. In addition, we did not find an effect of S. boulardii on SCFAs in kitten feces. Similarly, a study reported no significant effect of *S. boulardii* on SCFAs in the feces of breeding dogs [25]. Conversely, a recent study found that the addition of *S. boulardii* and *Pediococcus acidilactici* to feline diets increased the concentration of total SCFAs and butyric acid in cat feces, which may be beneficial for intestinal health [61]. Additionally, humanized mice supplemented with *S. boulardii* can increase the colonic contents of acetate, propionate, and butyrate, thereby alleviating colitis induced by dextran sulfate sodium [62]. As obligate carnivores, cats possess a distinctive intestinal structure and digestive system that enable them to efficiently digest and absorb high-protein and high-fat diets. Nevertheless, their capacity for fiber digestion is relatively poor [63,64]. Although some in vitro studies have shown that cats may increase the production of SCFAs by digesting fiber [65,66], current commercial cat foods are rich in protein and fat while limiting the use of fiber-rich ingredients. Similarly, only sweet potatoes, potatoes, and rice in this experimental diet could provide cats with a small amount of fiber. Most SCFAs are bacterial metabolites produced by specific anaerobic bacteria of the colon after the fermentation of dietary fiber. It is possible that the low fiber intake of experimental cats resulted in a lack of fermentable substrates for the gut microbiota to produce SCFAs. Therefore, the effects of enzymolysis seaweed powder and *S. boulardii* on SCFAs were nonsignificant under the test conditions.

The gut mucosal barrier is pivotal in preventing the invasion of pathogenic antigens and maintaining a normal intestinal function. Increased intestinal permeability indicates damage to the gut epithelial barrier, causing the penetration of toxins and pathogens [67]. D-LA and LPS are intestinal bacterial metabolites that disrupt the intestinal mucosal barrier, invade the bloodstream, and promote the release of inflammatory factors [68,69]. DAO is a highly active enzyme in the epithelial villi of the mammalian intestinal mucosa, and iFABP mainly exists in the mature intestinal epithelial cells of the small intestine. Under normal conditions, they are present at very low levels in the blood. Damage to the gut mucosal barrier increases permeability, resulting in the release of large quantities of the above substances into the bloodstream [70,71]. This study found that the dietary addition of enzymolysis seaweed powder significantly reduced plasma levels of D-LA, LPS, DAO, and iFABP in kittens compared with the basal diet and the *S. boulardii* diet. Consistent with our results, sodium alginate improved the gut mucosal barrier function in cyclophosphamide-induced immunosuppressed mice, which reduced serum concentrations of D-LA and LPS [72]. Furthermore, dietary supplementation with seaweed-derived polysaccharides could promote the levels of claudin-1, occludin, and ZO-1 in the jejunal mucosa and significantly reduced DAO activity and serum D-LA concentrations in weaned piglets [41]. Seaweed extract may improve intestinal health by strengthening the gut barrier function.

The gut is the largest immune organ of the body. Intestinal physical, chemical, and microbial barriers influence and alter host inflammation by regulating homeostasis as well as tolerating and preventing pathological immune responses. Cytokines, a class of endogenous polypeptides generated mainly by immune system cells, have many biological effects and can mediate various immune responses. The dynamic balance between pro-inflammatory and anti-inflammatory cytokines is the key for maintaining the body’s normal immune status and physiological activities [73]. IL-1β, IL-6, and TNF-α are typical pro-inflammatory cytokines. Among them, IL-1β and TNF-α are pleiotropic, and their local activation can lead to elevated levels of secondary inflammatory mediators including IL-6 [74]. Laminarin can significantly downregulate the expressions of colon mucosal inflammatory factors IL-6, IL-17, and IL-1β, thereby improving the intestinal health of piglets [75]. Additionally, the brown seaweed extract reduced IL-6, IL-8, and TNF-α expressions in piglets with LPS-induced colitis, thereby inhibiting the pro-inflammatory factor response [76]. Consistent with the findings of other studies, we found that a dietary addition of enzymolysis seaweed powder in kittens reduced TNF-α, IL-1β, and IL-6 levels, probably by inhibiting NF-κB activation to reduce the inflammatory response [77]. Seaweed polysaccharides were reported to inhibit NF-κB activation and downregulate the mRNA expressions of TLR-4, MyD88, and IκBα, thereby inhibiting IL-6 and TNF-α expressions [77]. IL-10 is a pleiotropic cytokine mainly secreted by antigen-presenting cells. It has powerful anti-inflammatory properties that inhibit the levels of pro-inflammatory cytokines by activating macrophages [78]. In our study, compared with CON diet- and *S. boulardii* diet-fed kittens, the serum IL-10 level in kittens fed with enzymatic seaweed powder significantly increased, indicating that enzymolysis seaweed powder improved the anti-inflammatory ability of kittens to some extent.

## 5. Conclusions

In this study, dietary supplementation with enzymolysis seaweed powder increased plasma immune and antioxidant capacities, improved intestinal barrier function, and reduced the inflammation level in kittens. In addition, the aforementioned diet enriched the abundance of Bacteroidetes, Lachnospiraceae, Prevotellaceae, Muribaculaceae, *Faecalibacterium*, *Bacteroides*, and *Eubacterium_hallii_group* in the guts of kittens. Our findings provide a basis for the application and value of enzymolysis seaweed powder in kitten food.

## Figures and Tables

**Table 1:** Effects of enzymolysis seaweed powder and *S. boulardii* on ADG and ADFI in kittens.

Items	CON	SE	SB	SEM	*p*-Value
BW, kg					
d 0	1.50	1.51	1.50	0.13	0.99
d 14	1.69	1.72	1.75	0.15	0.93
d 28	1.91	2.10	2.03	0.16	0.47
ADG, g/d					
d 0 to 14	13.6	15.3	17.9	2.93	0.35
d 15 to 28	15.6 ^b^	27.5 ^a^	19.6 ^b^	3.85	0.02
d 0 to 28	14.6 ^b^	21.4 ^a^	18.7 ^ab^	2.20	0.02
ADFI, g/d					
d 0 to 14	90.0	90.0	93.2	2.47	0.33
d 15 to 28	65.4	63.8	62.2	6.24	0.88
d 0 to 28	90.0	88.9	90.5	1.78	0.64

^a,b^ Within a row, means without a common superscript differ at *p* ≤ 0.05. CON, basal diet; SE, basal diet + enzymolysis seaweed powder (20 g/kg of feed); SB, basal diet + *S. boulardii* (2 × 10^10^ CFU/kg of feed). BW, body weight; ADG, average daily gain; ADFI, average daily feed intake.

**Table 2:** Effect of enzymolysis seaweed powder and *S. boulardii* on fecal scores in kittens.

Items	CON	SE	SB	SEM	*p*-Value
d 0	4.00	4.05	4.10	0.07	0.86
d 14	3.65 ^b^	2.75 ^a^	3.55 ^ab^	0.15	0.03
d 28	3.05 ^b^	2.20 ^a^	2.15 ^a^	0.10	<0.01

^a,b^ Within a row, means without a common superscript differ at *p* ≤ 0.05. CON, basal diet; SE, basal diet + enzymolysis seaweed powder (20 g/kg of feed); SB, basal diet + *S. boulardii* (2 × 10^10^ CFU/kg of feed).

**Table 3:** Effects of enzymolysis seaweed powder and *S. boulardii* on immunoglobulin and antioxidant parameters in kittens.

Items	CON	SE	SB	SEM	*p*-Value
IgA, μg/mL	177.4 ^c^	461.5 ^a^	387.9 ^b^	10.6	<0.01
IgG, μg/mL	2520 ^c^	5091 ^a^	4359 ^b^	138	<0.01
SOD, pg/mL	92.57 ^c^	220.6 ^a^	178.9 ^b^	5.48	<0.01
MDA, nmol/mL	8.602 ^a^	4.572 ^c^	5.954 ^b^	0.19	<0.01

^a–c^ Within a row, means without a common superscript differ at *p* ≤ 0.05. CON, basal diet; SE, basal diet + enzymolysis seaweed powder (20 g/kg of feed); SB, basal diet + *S. boulardii* (2 × 10^10^ CFU/kg of feed). IgA, immunoglobulin A; IgG, immunoglobulin G; SOD, superoxide dismutase; MDA, malondialdehyde.

**Table 4:** Effects of enzymolysis seaweed powder and *S. boulardii* on plasma inflammatory parameters in kittens.

Items	CON	SE	SB	SEM	*p*-Value
IL-1β, pg/mL	216.3 ^a^	149.9 ^c^	167.4 ^b^	3.78	<0.01
IL-6, pg/mL	38.91 ^a^	20.41 ^c^	26.17 ^b^	1.16	<0.01
IL-10, pg/mL	102.0 ^c^	219.2 ^a^	184.3 ^b^	6.12	<0.01
TNF-α, pg/mL	140.7 ^a^	88.11 ^c^	105.0 ^b^	2.75	<0.01

^a–c^ Within a row, means without a common superscript differ at *p* ≤ 0.05. CON, basal diet; SE, basal diet + enzymolysis seaweed powder (20 g/kg of feed); SB, basal diet + *S. boulardii* (2 × 10^10^ CFU/kg of feed). IL-1β, interleukin-1β; IL-6, interleukin-6; IL-10, interleukin-10; TNF-α, tumor necrosis factor-α.

**Table 5:** Effects of enzymolysis seaweed powder and *S. boulardii* on plasma intestinal barrier function parameters in kittens.

Items	CON	SE	SB	SEM	*p*-Value
D-LA, μmol/L	145.3 ^a^	98.54 ^c^	113.8 ^b^	2.44	<0.01
LPS, EU/L	873.6 ^a^	363.3 ^c^	496.8 ^b^	24.6	<0.01
DAO, ng/mL	56.06 ^a^	32.32 ^c^	41.57 ^b^	0.82	<0.01
iFABP, pg/mL	1842 ^a^	1130 ^c^	1392 ^b^	32.2	<0.01

^a–c^ Within a row, means without a common superscript differ at *p* ≤ 0.05. CON, basal diet; SE, basal diet + enzymolysis seaweed powder (20 g/kg of feed); SB, basal diet + *S. boulardii* (2 × 10^10^ CFU/kg of feed). D-LA, D-lactate; LPS, lipopolysaccharide; DAO, Diamine oxidase; iFABP, intestinal fatty acid-binding protein.

**Table 6:** Effects of enzymolysis seaweed powder and *S. boulardii* on SCFA concentrations in kittens.

Items, mg/kg	CON	SE	SB	SEM	*p*-Value
Acetate	3280	3609	3634	324.3	0.49
Propionate	2792	2782	2494	423.5	0.73
Butyrate	2247	2049	2158	360.3	0.86
Isobutyrate	280.5	240.2	238.9	51.71	0.66
Valerate	1027	1099	1063	194.1	0.93
Isovalerate	461.5	528.7	445.4	96.11	0.66

CON, basal diet; SE, basal diet + enzymolysis seaweed powder (20 g/kg of feed); SB, basal diet + *S. boulardii* (2 × 10^10^ CFU/kg of feed).

## Data Availability

For this study, data are available from the corresponding authors upon request. The data are not publicly available due to the related product is under development and patent application.

## Appendix A

**Table A1:** Ingredient composition and nutrient levels of the experimental diets (as-fed basis).

Items	Content
Ingredients, %	
Chicken meal	46.25
Potato starch	19.00
Poultry fat	9.50
Fish meal	5.50
Chicken livers	4.50
Rice	4.00
Potato	3.00
Fish oil	3.00
Sweet potato	3.50
Salt	0.45
Taurine	0.15
Choline chloride	0.40
Magnesium sulfate	0.25
Vitamin Premix ^1^	0.30
Mineral premix ^2^	0.20
Total	100.00
Analyzed nutrient levels ^3^	
Dry matter, %	90.61
Total energy, MJ/kg	23.02
Crude protein, %	39.98
Ether extract, %	22.39
Ash, %	7.98

Note: ^1^ Vitamin premix provided the following per kilogram of feed: vitamin A (15,000 IU), vitamin B_1_ (30 mg), vitamin B_2_ (28 mg), vitamin B_3_ (110 mg), vitamin B_5_ (85 mg), vitamin B_6_ (12 mg), vitamin B_12_ (0.19 mg), vitamin D_3_ (15 IU), and vitamin E (75,300 IU); ^2^ Mineral premix provided the following per kilogram of feed: Ca (CaI_2_) 20 mg, Co (CoSO_4_) 0.10 mg, Cu (CuSO_4_) 3 mg, Fe (FeSO_4_) 50 mg, I (CaI_2_) 40 mg, Mn (MnSO_4_) 18 mg, Na (Na_2_SeO_3_) 0.05 mg, Zn (ZnSO_4_) 38 mg, Se (Na_2_SeO_3_) 260 mg; ^3^ The nutrient levels of the diets were analyzed.
